# The functional mechanisms of phosphite and its applications in crop plants

**DOI:** 10.3389/fpls.2025.1538596

**Published:** 2025-04-07

**Authors:** Zhenyi Li, Xiangjiu Kong, Zhiqiang Zhang, Fang Tang, Mingjiu Wang, Yan Zhao, Fengling Shi

**Affiliations:** ^1^ Key Laboratory of Grassland Resources, Ministry of Education People's Republic of China, College of Grassland Science, Inner Mongolia Agricultural University, Hohhot, China; ^2^ College of Horticulture and Plant Protection, Inner Mongolia Agricultural University, Hohhot, China; ^3^ National Center of Pratacultural Technology Innovation (under preparation), Hohhot, China

**Keywords:** phosphite, fungicide, herbicide, bio-stimulant, alkaline phosphatase, phosphite dehydrogenase

## Abstract

Phosphite (Phi), the reduced form of phosphate (Pi), is characterized by its stability, high solubility, efficient transport, resistance to fixation in soil, and widespread occurrence in natural environments. Although Phi exhibits greater suitability than Pi as a soil fertilizer, it cannot be metabolized by plants. In agricultural applications, Phi serves as a bio-stimulant, fungicide, herbicide, and has other purposes. As a bio-stimulant, Phi has been shown to promote plant growth, enhance stress resistance, and improve fruit quality. Additionally, when used as a fungicide or pesticide, it effectively inhibits the growth of phytopathogens in various crop species. The discovery of the *phosphite dehydrogenase* (*ptxD*) gene in microorganisms has significantly expanded the potential applications of Phi, including its use as a herbicide, phosphatic fertilizer, and a selectable chemical for generating marker-free transgenic plants. Therefore, the dual fertilization and weed control system of *ptxD*/Phi facilitates the utilization of Phi as the sole phosphorus source while concurrently suppressing the evolution of herbicide-resistant weeds in the future. Notably, *ptxD* also acts as an ideal selectable marker because its resistant is specific to Phi, thereby eliminating the risk of false positive clones. The application of Phi provides a promising strategy for addressing phosphorus resource shortages and improving the efficiency of phosphatic fertilizers in agriculture. Furthermore, Phi is considered an environmentally friendly fertilizer, as it contributes to the mitigation of eutrophication. In prospect, Phi is anticipated to play a significant role as a chemical fertilizer that promotes the sustainable development of agriculture. In this review, we provide a comprehensive analysis of the functional mechanisms of Phi and its current applications in agriculture, with the aim of offering deeper insights into its potential benefits and practical utility.

Phosphite (Phi) is a reduced form of phosphate (Pi). One oxygen atom of Pi is replaced by a hydrogen atom in Phi (Liu et al., 2023), rendering Phi a kinetically stable and highly soluble compound in soil. Phi mainly exists in soil (Kehler et al., 2021), freshwater (Han et al., 2013), marshes (Pasek et al., 2014), sediments (Tapia-Torres et al., 2016), and oceans (Mooy et al., 2015) in various oxidation states, accountings for 10%–30% of all phosphorus (P) compounds on Earth (Figueroa and Coates, 2017). It has been integrated into the soil for nearly one century and is mainly released into the soil during mining and utilization of Pi rocks (Liu et al., 2023). With the advancement of industrialization, Phi accumulates in soils through various industrial and agricultural pathways. For example, Phi is released into the environment during the production and utilization of Pi-based products such as organophosphorus fungicides and elemental P (Figueroa and Coates, 2017; Liu et al., 2023). In addition, Phi is commonly generated through ferrous oxidation and microbial transformation processes (Ewens et al., 2021; Figueroa and Coates, 2017; Liu et al., 2023). Phi is also a byproduct of the electronics, automobile, pharmaceutical, chemical, and construction industries, where it is mainly used as a reducing agent in processes such as nickel plating and polishing (Heuer et al., 2017). Notably, the accumulation of Phi in soils is primarily attributed to agricultural inputs, which are subsequently transported into lakes and wetland systems through rain water runoff (Liu et al., 2023).

In recent years, Phi has gained increasing attention and is widely used as a fertilizer, bio-stimulant, fungicide, herbicide, and selectable marker for transgenic plants (Achary et al., 2017; Gómez-Merino et al., 2022; Gómez-Merino and Trejo-Téllez, 2015; Havlin and Schlegel, 2021; Heuer et al., 2017; Manna et al., 2016) (**Figure 1**). The application of Phi contributes to alleviating P shortages, reducing the evolution of super-weeds, mitigating eutrophication, and providing various other benefits (**Figure 1**). Phi is reported to have low environmental toxicity and poses minimal risk to animals and humans (Achary et al., 2017; Havlin and Schlegel, 2021; Lobato et al., 2010). However, the health and environmental impacts of the maximum residue levels (MRLs) of Phi should be carefully examined in the context of agricultural production (Gómez-Merino et al., 2022). In the United States, Phi is not regulated under food administration guidelines (Aćimović et al., 2016). In contrast, the European Union maximum has established allowable Phi MRLs range from 2–80 ppm, depending on the crop category (Estrada-Ortiz et al., 2016).

**Figure 1:**
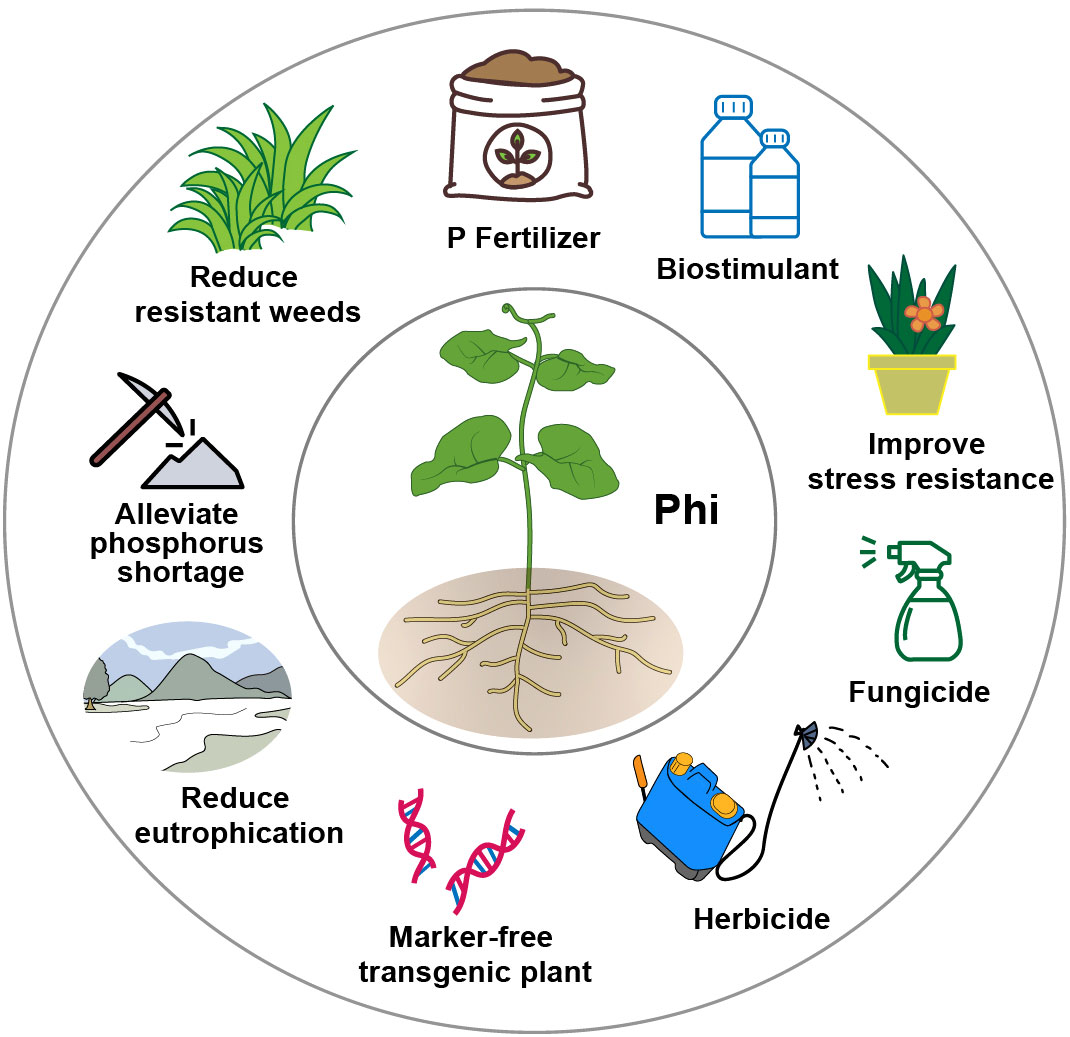
The utilization and potential advantages of phosphite (Phi) in agriculture.

## 1 Characteristics of Phi and its absorption in plants

Phi, also referred to as phosphoric acid or phosphonate, is structurally similar to Pi. The P atom in Pi (PO_4_^3+^) has a valence of +5, and is primarily assimilated in plants in the forms of H_2_PO_4_^-^ and HPO_4_^2-^ forms. In contrast, the P atom in Phi (PO_3_^3+^) has a valence of +3, and is assimilated in plants in the forms of H_2_PO_3_^-^ and HPO_3_^2-^ forms (Gómez-Merino et al., 2022; Havlin and Schlegel, 2021). Compared to Pi, Phi exhibits greater stability, has high transport efficiency, and solubility, and is less prone to fixation. Phi is also unable largely inaccessible to be used by most microorganisms and demonstrates solubility that is 100 times greater than Pi (Danova-Alt et al., 2008; Figueroa and Coates, 2017; Herrera-Estrella and Lopez-Arredondo, 2016; Heuer et al., 2017; Jost et al., 2015; McDonald et al., 2001a; White and Metcalf, 2007). Moreover, the kinetic stability of Phi minimizes its involvement in unnecessary chemical reactions.

**Figure 2:**
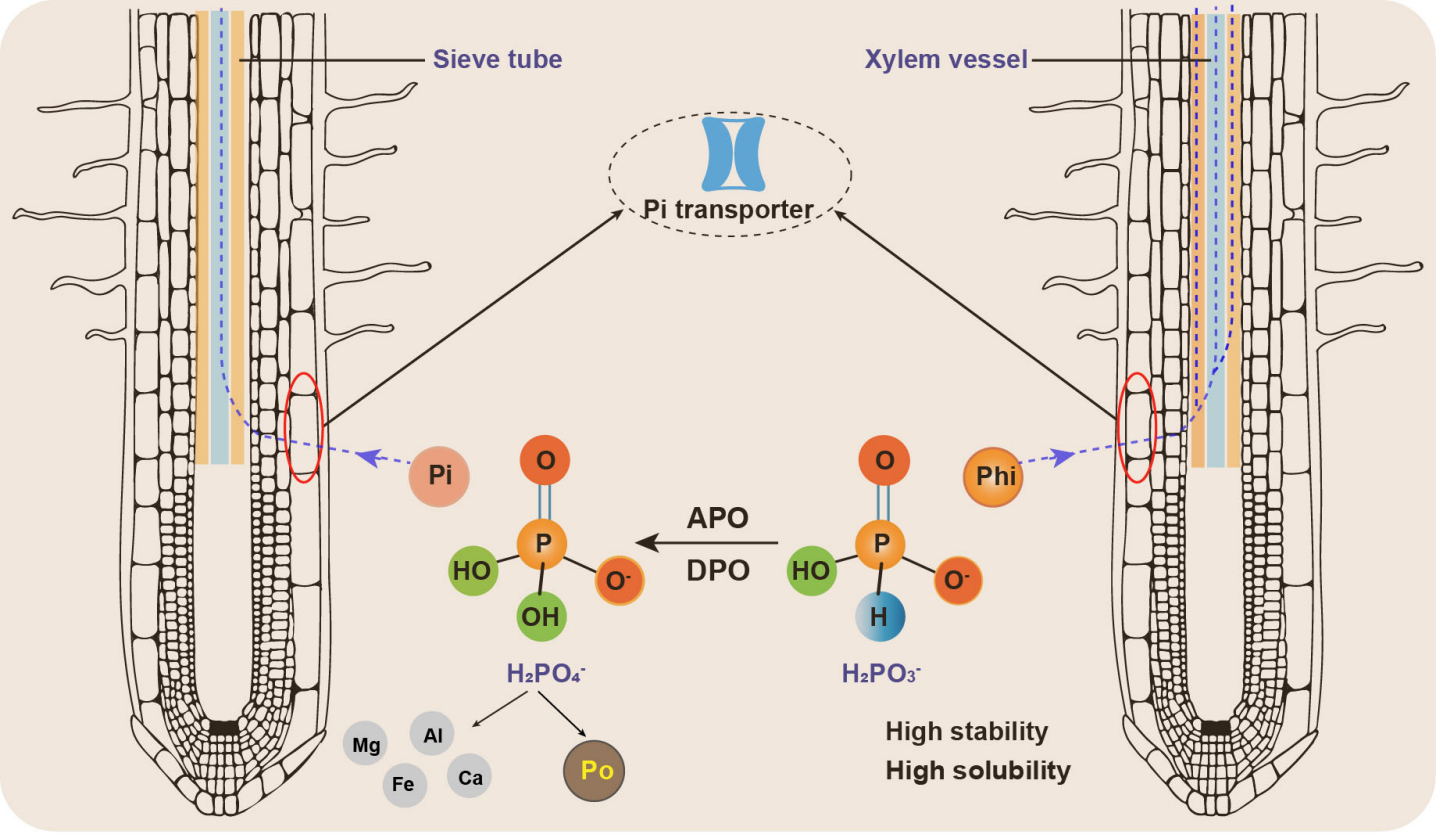
The differences in characteristics and exhibition of phosphite and phosphate in soil. The abbreviations are as follows. Phi, phosphite; Pi, inorganic phosphate; Po, organic phosphorous; APO, assimilatory phosphite oxidation; DPO, dissimilatory phosphite oxidation.

Some studies suggest that plant cells may uptake Phi more rapidly than Pi. The presence of three oxygen atoms in the Phi molecule allows its transport through both the xylem and phloem (Jost et al., 2015; McDonald et al., 2001b) (**Figure 2**), whereas Pi is only transported through the xylem. Phi uptake is pH-dependent and competes with Pi for absorption (Ouimette and Coffey, 1990). Both Phi and Pi are acquired by the same transport system, which includes high-affinity and low-affinity Pi transporters (Achary et al., 2017). Phi can be absorbed through the roots and leaves and is primarily stored in the cytoplasm and vacuoles (Danova-Alt et al., 2008).

The main difference between Pi and Phi is that Pi is readily converted to organic P molecules immediately upon absorption, whereas Phi is not (Guest and Grant, 1991). Despite being easily absorbed and transported by plants, Phi cannot be oxidized or metabolized (Havlin and Schlegel, 2021). Thus, Phi impedes the growth and metabolism of Pi-deficient plants by suppressing their molecular and developmental responses to phosphate starvation (Varadarajan et al., 2002). Phi also exhibits a higher efficacy in the soil, allowing it to dissolve and be assimilated without requiring large amounts of energy (Achary et al., 2017). Furthermore, Phi functions in optimal synergy with other nutrients, such as potassium (K), calcium (Ca), boron (B), zinc (Zn), molybdenum (Mo) and manganese (Mn), making it a long-considered ideal phosphatic fertilizer (Havlin and Schlegel, 2021; Herrera-Estrella and Lopez-Arredondo, 2016).

## 2 Functional mechanisms of Phi-influenced plants

### 2.1 Phi inhibited plant growth>

Phi exhibits toxicity at high concentrations but exerts beneficial effects on plants at low concentrations (Estrada-Ortiz et al., 2016; Trejo-Téllez and Gómez-Merino, 2018). The inhibitory effects of Phi on Plants occur via two mechanisms. First, the accumulation and toxic effects of Phi are likely associated with reduced Pi assimilation and the inability to metabolize Phi or convert it Pi within the cells (Thao and Yamakawa, 2009; Ticconi et al., 2001). Second, Phi attenuates the phosphate starvation response (PSR) in cells, reducing the plant’s sensitivity to P deficiency (Ticconi et al., 2001). Phi suppresses the activity of key enzymes implicated in the PSR, including ATPase and PPI-dependent phosphofructokinase. This inhibition diminishes the PSR, particularly in plants suffering from inorganic phosphate (Pi) deficiency.

In Pi-deficient soils, Phi may be perceived by plants as Pi, thereby inhibiting the activation of P starvation responses that are critical for maintaining plant growth and function (McDonald et al., 2001a). However, Phi cannot participate in Pi-related biological processes due to its inability to be oxidized to Pi (Achary et al., 2017; Danova-Alt et al., 2008). As a result, Phi stress leads to arrested plant growth and the development of and toxic symptoms (Havlin and Schlegel, 2021; Singh et al., 2003; Thao et al., 2008a; Ticconi et al., 2001; Zambrosi et al., 2011). Plants can absorb Phi due to its structural similarity to Pi (McDonald et al., 2001b). However, Pi antagonistically inhibits the uptake of Phi in plants (Jost et al., 2015; Pratt et al., 2009; Trejo-Téllez and Gómez-Merino, 2018). In *Phytophthora*, Pi and Phi compete for the binding sites of Pi transporters (Griffith et al., 1989). Under Pi-deficient conditions, Phi accumulates in the cytoplasm, but when Pi is supplied, Phi is rapidly expelled from the cell (Achary et al., 2017). The presence of Pi enhances the concealed role of Phi in the vacuole. Under adequate Pi conditions, plants tolerate moderate concentrations of Phi without adverse effects (Thao and Yamakawa, 2009). However, under low-Pi conditions, Phi blocks the Pi starvation-induced transduction signaling pathway, weakening the cellular response to P deficiency and impairing P utilization even when Pi is sufficient (Singh et al., 2003; Ticconi et al., 2001; Vinas et al., 2020).

Phi functions in metabolic interruption and growth inhibition by suppressing phosphorylation and competing for the Pi-binding sites of phosphorylase (Achary et al., 2017). Phi stress causes defective phenotypes in plants, such as stunted growth of primary roots, yellowing of young leaves, and patchy anthocyanin accumulation in old leaves (Hirosse et al., 2012; Manna et al., 2015; Thao et al., 2008a, b). In common bean (*Phaseolus vulgaris*), Phi inhibits growth, resulting in poor grain filling under Pi-deficient conditions (Ávila et al., 2013). Similarly, in potato (*Solanum tuberosum*), Phi significantly reduces shoot length, root length, total root length, root volume, root tips, and fresh biomass (Dormatey, 2022). Phi also inhibits photosynthesis and the TCA cycle in the aboveground parts of alfalfa (*Medicago sativa*) seedlings (Li et al., 2022). The growth rate, length, and dry weight of sweet potato (*Ipomoea batatas*) are significantly reduced by excessive Phi application (Hirosse et al., 2012). In addition, the shoot dry weight and P concentration of spinach (*Spinacia oleracea*) decrease as the Pi: Phi ratio decrease from 100:0 to 0:100 (Thao et al., 2008a). Phi also significantly inhibits root growth and root hair development in both onion (*Allium cepa*) (Carswell et al., 1996) and spinach and reduces biomass and chlorophyll content in citrus (*Citrus* spp.) (Zambrosi et al., 2011).

### 2.2 Phi promotes plant growth

Phi has been shown to enhance plant growth and development (Gómez-Merino et al., 2022; Gómez-Merino and Trejo-Téllez, 2015; Thao and Yamakawa, 2009; Trejo-Téllez and Gómez-Merino, 2018). Several hypotheses have been proposed regarding its functional mechanism in promoting plant growth (Gómez-Merino et al., 2022). Phi influences sugar metabolism, modulates plant hormones levels, and impacts secondary metabolite synthesis by inducing the shikimic acid pathway (Lovatt and Mikkelsen, 2006). The shikimic acid pathway is responsible for the biosynthesis of aromatic amino acids such as phenylalanine, tyrosine, and tryptophan. These amino acids serve as precursors for a diverse array of secondary metabolites, including pigments, alkaloids, hormones, and cell wall components, which are crucial for plant growth and development (Gómez-Merino and Trejo-Téllez, 2015). Phi treatment enhances the levels of free amino acids, proteins, sugars, and anthocyanins in strawberry leaves (Estrada-Ortiz et al., 2013). Phi promotes plant growth by upregulating genes associated with the biosynthesis and signaling pathways of abscisic acid (ABA), salicylic acid (SA), and jasmonic acid (JA) (Pérez-Zavala et al., 2024). Moreover, Phi improves fruit quality by promoting the synthesis of ascorbic acid and anthocyanins (Gómez-Merino and Trejo-Téllez, 2015). Notably, when combined coupled with metal ions, Phi facilitates faster and more nutrient delivery within plants, thereby exhibiting significant fertilizer effects (Havlin and Schlegel, 2021; Herrera-Estrella and Lopez-Arredondo, 2016; Lovatt and Mikkelsen, 2006).

Studies have reported Phi enhances crop flowering, yield, quality, fruit size, and soluble matter content, functioning effectively as a bio-stimulant (Gómez-Merino et al., 2022; Gómez-Merino and Trejo-Téllez, 2015; Martínez, 2016). Currently, Phi is widely utilized to supplement plant nutrition and optimize agricultural productivity for growers (Havlin and Schlegel, 2021). For instance, it enhances the yield and quality of rice (*Oryza sativa*) (Martínez, 2016), soybean (*Glycine max*) (Carmona et al., 2018), strawberry (*Fragaria × ananassa*) (Estrada-Ortiz et al., 2013; Glinicki et al., 2010), onion bulb (*Allium cepa*) (Monsalve et al., 2012), squash (*cucurbita pepo*) (Omar et al., 2020), celery (*Apium graveolens*), peach (*Prunus persica*), and sweet orange (*Citrus sinensis*) and improves flowering in potato and tomato (*Solanum lycopersicum*) (Lovatt and Mikkelsen, 2006; Rickard, 2000). Foliar spray application of 1,775-3,550 g/hm^2^ potassium (K) Phi increased rice yield by 5%-10% (Martínez, 2016). Seed treatment with K- and Mn-Phi increased seedling emergence by up to 29% compared to control (Carmona et al., 2018). In addition, fertigation with either sole Phi (consisting of 6.7% Phi of P) or Phi combined NPK increased strawberry shoot and root growth (Glinicki et al., 2010). Hydroponic application of 20%-30% Phi improved fruit quality by increasing anthocyanin concentrations in strawberry (Estrada-Ortiz et al., 2013). Notably, a nutrient solution containing 50% H_3_PO_4_ and 50% H_3_PO_3_ enhanced biomass, leaf area, and total P content in lettuce, tomato, and banana (*Musa paradisiaca*) (Bertsch et al., 2009). Foliar spray application of 5 mL/L potassium Phi efficiently increased the weight of first-class onion bulbs and total bulb weight (Monsalve et al., 2012). Potatoes treated with potassium Phi have a shorter interval between planting and germination and have increased leaf area and weight (Tambascio et al., 2014). Therefore, the optimal concentration of Phi, in combination with other essential nutrient ions, represents a promising bio-stimulant and a transformative enhancer of crop production and quality in modern agricultural systems.

Notably, the availability of Pi to plants is a crucial determinant of Phi toxicity. Co-application of Pi and Phi synergistically improved P absorption, confirming that Phi toxicity is proportional to Pi utilization (Bertsch et al., 2009). In contrast, exclusive Phi application inhibited growth, causing leaf wilting and root degeneration (Bertsch et al., 2009; Gómez-Merino and Trejo-Téllez, 2015). The effects of Phi on the metabolism of beets and lettuce varied different under hydroponic conditions. Specifically, Phi caused positive responses, including increased biomass and nutrient content, under sufficient-P conditions and at concentrations is less than 0.25 mM (Estrada-Ortiz et al., 2016).

However, Phi takes approximately four months to be fully oxidized to Pi in the natural environment (McDonald et al., 2001a), making it an ideal candidate for use as a slow-release fertilizer. Despite the challenges associated with promoting Phi in agricultural production, it holds significant potential to increase crop yields and health, address the challenges of feeding a growing global population, and minimize the negative impacts of agriculture on human health and the environment (Han et al., 2021). Therefore, Phi-based fertilizers are poised for development and could serve as a viable alternative to conventional P fertilizers in the future.

### 2.3 Phi-induced resistance in plants to abiotic stresses

Phi effectively improves plant tolerance to diverse abiotic stresses, including UV radiation, water deficit, and heat shock (Gómez-Merino and Trejo-Téllez, 2015; Trejo-Téllez and Gómez-Merino, 2018; Xi et al., 2020). Phi activates defense responses against pathogens and modulates primary metabolism to help plants cope with abiotic stress. Furthermore, dual-channel Phi transport reduces energy consumption during nutrient transport, thereby providing additional energy to mitigate the effects of abiotic stress. This induces the accumulation of proteins associated with cell wall formation in plants subjected to abiotic stress (Trejo-Téllez and Gómez-Merino, 2018). In potatoes, Phi induces systemic defense response, including an increase in the levels of phytoantibiotics and chitinase, as well as enhanced activities of peroxidase and polyphenol oxidase (Lobato et al., 2011). Phi also improves the plant’s tolerance to UV stress by activating the antioxidant system and inducing the natural defense response of plants (Soledad et al., 2015). Pre-treatment with Phi enhanced potato resistance to UV-B, as evidenced by increased chlorophyll accumulation and elevated expression of the *D1 polypeptide-encoding gene* (*psbA*) of the photosystem II in the chloroplast, which serves as a photosynthetic protection protein (Soledad et al., 2015). Application of 0.25 mM Phi in hydroponic solutions increased P and chlorophyll concentrations in lettuce (*Lactuca sativa*) (Estrada-Ortiz et al., 2016). Phi also enhanced pathogen tolerance by improving oxidative levels, including SOD, POX, CAT, and APX activities, along with higher concentrations of antioxidant metabolites such as phenolics, flavonoids, and proline and proteins and carbohydrates in potato leaves (Mohammadi et al., 2020). In alfalfa Phi treatment increased the abundance of *heat shock protein* (*HSP*), *mitochondrial alternative oxidase* (*AOX*), and *pathogenicity-related protein* (*PR protein*) as well as enhanced DNA repair through enzymatic and non-enzymatic oxidative systems (Li et al., 2022).

Pretreatment with a Phi solution before planting effectively activates the defense response in fruits, increasing the content of ascorbic acid and anthocyanins (Estrada-Ortiz et al., 2013; Moor et al., 2009). The synthesis and accumulation of anthocyanins play a key role in responding to nutrient deficiency and pathogen infection (Routray and Orsat, 2011). Anthocyanins act as light attenuators, reducing photo-oxidative damage to leaves by shielding chloroplasts from excessive high-energy ions and scavenging reactive oxygen species (Zheng et al., 2021). Additionally, Phi promotes the synthesis of antioxidant enzymes and metabolites (Ávila et al., 2013). Low concentrations of Phi increase catalase activity, while medium and high levels of Phi significantly reduce enzyme activity under P deficiency (Ávila et al., 2013). Plants experiencing either Pi deficiency or excessive Phi exhibit reduced growth and diminished tolerance to toxicity (Trejo-Téllez and Gómez-Merino, 2018). These complex phenomena are attributed to the underlying overlapping of signaling pathways and their intricate interactions (Gómez-Merino and Trejo-Téllez, 2015).

## 3 Mechanism and application of Phi response to disease

### 3.1 Functional mechanism of Phi response to disease

Phi is highly effective in controlling a wide range of phytopathogens, including pathogenic bacteria, oomycetes, fungi, and nematodes (Grant et al., 1992; Lobato et al., 2010). Phi or chemical formulations containing Phi-active ingredients are used to control various plant pathogens by activating the plant defense system (Thao et al., 2008b). Although Phi has demonstrated a beneficial role in enhancing plant tolerance to biotic stress, the underlying mechanisms of its response remain unclear. Based on various experimental studies, numerous discussions have focused on how plants respond to biotic stress in the presence of Phi. (1) Phi directly targets pathogens and induces plants to produce inhibitors of toxic substances (Thao et al., 2008b). For example, Phi-induced disease resistance proteins rely on the salicylic acid (SA) pathway to elicit defensive effects (Havlin and Schlegel, 2021). While it is primarily hypothesized that Phi acts indirectly on plant diseases, it can also directly enhance plant health by controlling specific fungi on cultivated or wild plants. Overall, Phi serves as an initiator of multiple plant defense responses (Havlin and Schlegel, 2021) and operates through a complex mechanism to prevent oomycete infections. (2) Phi directly increases cell wall thickness, preventing further invasion and expelling pathogens from plant tissues (Carswell et al., 1996). It inhibits hyphae growth and spore germination, and suppresses or modulates membrane metabolism and phosphorylation reactions of pathogens. Phi also acts indirectly by activating plant defense responses (Daniel and Guest, 2005). By disrupting cellular sugar metabolism, Phi induces a state of sugar deprivation within plant cells, which stimulates the expression of chitinase genes, leading to the continuous degradation of newly synthesized chitin at the tips of mycelia (Andreu et al., 2006; Yang et al., 2006). Notably, the complexity of this defense mechanism prevents pathogens from developing resistance to these inhibitory effects.

### 3.2 Application of phi as fungicide and insecticide

In agricultural production, Phi has been shown to effectively control pathogens in over 20 crops, including rice (Huang et al., 2020; Martínez, 2016), corn (Dias-Arieira et al., 2012), wheat (Oka et al., 2007), soybean (Dias-Arieira et al., 2012; Silva et al., 2011), common bean (Fagundes-Nacarath et al., 2018), potato (Borza et al., 2014; Burra et al., 2014; Lobato et al., 2011; MaChinandiarena et al., 2012), tomato (Su et al., 2022), strawberry (Marin et al., 2023), and grape (Speiser et al., 2000). The diseases controlled mainly include downy mildew, late blight, root rot, white mold, dieback, bacterial wilt, canker and others. It is worth noting that Phi exhibits varying effects against different pathogens across different crops. **Table 1** outlines the optimal concentrations of Phi for controlling various crop diseases.

**Table 1:** The effect of Phi-controlled fungi- and nematode-induced diseases in different plants.

Plant	Disease	Fungi or nematode	Phosphite treatment	Reference
rice	stem and sheath disease	*Nakataea oryzae*	KPhi combined with strobilurin and triazole	(Martínez 2016)
	Phytophthora disease	*Xanthomonas oryzae* pv. oryzae and *Pyricularia grisea*	50 ppm phosphite	(Huang et al., 2020)
maize	downy mildew	*Peronosclerospora sorghi*	phosphonic acid (20%) neutralized with an equal amount of KOH	(Panicker and Gangadharan 1999)
wheat	nematodes	*Heterodera avenae* and *Meloidogyne marylandi*	0.63 mg of phosphite (HPO_3_^2–^) per plant	(Oka et al., 2007)
maize and soybean	nematode	*Pratylenchus brachyurus*	1.5 mL/L KPhi	(Dias-Arieira et al., 2012)
soybean	downy mildew	*Peronospora manshurica*	375 g/hm^2^ KPhi	(Silva et al., 2011)
	root rot	*Phytophthora sojae*	4.5 mg/ml KPhi	(Guo et al., 2021)
	pythium damping-off	*Pythium aphanidermatum* (Edson) Fitzpatrick*, Pythium irregulare Buisman*, and *Pythium ultimum*	400 mL KPhi per seed, 400 mL MnPhi for 100 kg seed treatment	(Carmona et al., 2018)
common bean	anthracnose	*Colletotrichum lindemuthianum*	4 mL/L KPhi	(Figueira et al., 2020)
	white mold	*Sclerotinia sclerotiorum*	5 mL/L ZnPhi or 2.5 mL/L CuPhi	(Fagundes-Nacarath et al., 2018)
lupin	dieback	*Phytophthora cinnamomi*	10 kg/hm^2^ KPhi	(Smillie et al., 1989)
tomato	bacterial wilt	*Ralstonia solanacearum*	0.05% (wt/vol) KPhi	(Su et al., 2022)
potato	late blight	*Phytophthora infestans, Rhizoctonia solani, Fusarium solani* and *Streptomyces scabies*		(Lobato et al., 2011)
	late blight	*Phytophthora infestans*	5.3 kg/hm^2^ KPhi; 3 L/hm^2^ 1% KPhi; 36 mM KPhi	(Borza et al., 2014) (Machinandiarena et al., 2012) (Burra et al., 2014)
	late blight	*Phytophthora infestans, Fusarium solani, Rhizoctonia solani, Streptomyces scabies*	1% or 67% CaPhi	(Lobato et al., 2010)
	late blight	*Phytophthora infestans*	2.5 L/hm^2^ KPhi and 0.2 L/hm^2^ Shirlan	(Liljeroth et al., 2016)
	late blight	*Phytophtora infestans*	3 L/hm^2^ 5 g/L KPhi	(Mohammadi et al., 2020)
	root and stem rot	*Phytophthora cinnamomi*	5 μg/mL	(Coffey and Joseph 1985)
*Arabidopsis thaliana*	phytophthora	*Phytophthora cinnamomi*	20 mM KPhi	(Eshraghi et al., 2011)
strawberry	crown rot and leather rot	*Phytophthora cactorum*	300 μg/mL 56% KPhi	(Marin et al., 2023)
cucumber	damping-off	*Pythium ultimum*	none	(Abbasi and Lazarovits 2006)
grape	downy mildew	*Plasmopara viticola*	10.45% phosphonate	(Speiser et al., 2000)
*Pinus radiata*	pitch canker	*Fusarium circinatum*	1% phosphite	(Cerqueira et al., 2017)
*Banksia grandis* and *Eucalyptus marginata*		*Phytophthora cinnamomi*	5 g/L to 10 g/L phosphite	(Wilkinson et al., 2001)

KPhi represents potassium phosphite, MnPhi represents manganese phosphite, ZnPhi represents zinc phosphite, CuPhi represents copper phosphite. The plants and its latin names are as follows: rice (*Oryza sativa*), maize (*Zea mays*), soybean (*Glycine max*), common bean (*Phaseolus vulgaris*), lupin (*Lupinus angustifoliu*), tomato (*Solanum lycopersicum*), potato (*Ipomoea batatas*), strawberry (*Fragaria × ananassa*), cucumber (*Cucumis sativus*) and grape (*Vitis vinifera*).

Phi effectively reduced potato tuber disease symptoms caused by *Phytophthora*, *Fusarium wilt*, and *Rhizoctonia solani* (Lobato et al., 2010). Copper phosphite (CuPhi) significantly resisted four pathogens, including *Phytophthora infestans*, *Fusarium solani*, *Rhizoctonia solani*, and *Streptomyces scabies*. Similarly, calcium Phi (CaPhi) and potassium Phi (KPhi) exhibited comparable capability in defending against pathogens (Lobato et al., 2010). In potatoes, 1% and 0.67% potassium Phi inhibited the growth of *Streptomyces* by nearly 80% and 60%, respectively (Lobato et al., 2010). Potassium Phi has been used in combination with the biological control agent *Bacillus amyloliquefaciens* OPF8 (strain F8) to control bacterial wilt in tomatoes (Su et al., 2022). A 0.05% concentration of KPhi significantly inhibited the growth of *Ralstonia solanacearum* (Su et al., 2022). Similarly, 0.05% K-Phi enhanced plants’ resistance to *B. amyloliquefaciens* F8 (Su et al., 2022). Martínez (2016) reported that potassium Phi, when combined with fungicides, strobilurin, and triazole, effectively inhibited rice stem rot caused by *Nakataea oryzae* (Martínez, 2016). In a large-scale field trial, Liljeroth et al. (2016) found that combining KPhi with half-dose fluoscymidone (0.2 L/hm^2^) was more effective in protecting against late blight in potatoes (Liljeroth et al., 2016). Phi is highly effective in controlling avocado (*Persea americana*) root rot and stem rot. However, long-term use of Phi resulted in some degree of resistance. For example, the roots of young *Lupinus angustifolius* seedlings were more susceptible to colonization by the Phi-resistant *Phytophthora cinnamomi* strain, which produced more cysts and zoospores compared to sensitive strains (Hunter et al., 2023). KPhi inhibited *Phytophthora cinnamomi* spore formation on *Banksia grandis* and *Eucalyptus marginata* in greenhouses (Wilkinson et al., 2001). Moreover, potassium Phi effectively reduced the production of zoospores on susceptible plants (Wilkinson et al., 2001).

Phi is mainly applied through foliar spraying. Other application methods include root irrigation, drip irrigation, hydroponic nutrient solution mixing, and immersion treatment (Achary et al., 2017). Foliar spraying of KPhi on winter wheat effectively reduced the occurrence of snow plum leaf blight and yellow sickle pathogens (Hofgaard et al., 2010). Phi spraying also significantly reduced the incidence and severity of scab on walnut leaves and fruits (Clive H. Bock et al., 2012). In addition, foliar spraying of Phi effectively mitigated the severity of late blight on potato tubers (Singh et al., 2003) and decreased the damage caused by fungal downy mildew in soybeans (Silva et al., 2011). Dias-Arieira et al. (2012) reported that potassium Phi effectively reduced the population of short-bodied nematodes (*Pratylenchus brachyurus*) by stimulating plant defense mechanisms, including phytoalexin production (Dias-Arieira et al., 2012).

In recent years, Phi-based fungicides have dominated the market. Notably, Bayer Crop Science has developed two globally recognized brands, Aliette and Fosetyl-Al, both of which feature Phi as their active ingredient. Other manufacturers also offer Phi-based fungicides that contain potassium, ammonium (NH_4_^+^), sodium (Na), and aluminum (Al) under various commercial brands (Achary et al., 2017). In 2023, the global market value of potassium Phi fungicides was reported to be $107 million (Market Reports World Global Potassium Phosphite Market—Market Reports World). Given Phi’s disease prevention properties, its market competitiveness has significantly increased, and it is anticipated that Phi fertilizers will be extensively utilized in future agricultural practices.

## 4 Mechanism and application of Phi as fertilizer and herbicide

### 4.1 Mechanism of Phi oxidization to Pi

In nature, the transformation of Phi to Pi through Phi oxidation and Pi reduction depends on the dominant microbial populations. However, Pi reduction is less commonly reported compared to Phi oxidation. Phi oxidation is an energy-efficient process, and some microorganisms oxidize Phi to Pi, subsequently using it as a source of P for cellular uptake (Ewens et al., 2021; Liu et al., 2023). The pathways of Phi oxidation include assimilatory phosphite oxidation (APO) and dissimilatory phosphite oxidation (DPO) (Figueroa and Coates, 2017; Liu et al., 2023).

**Figure 3:**
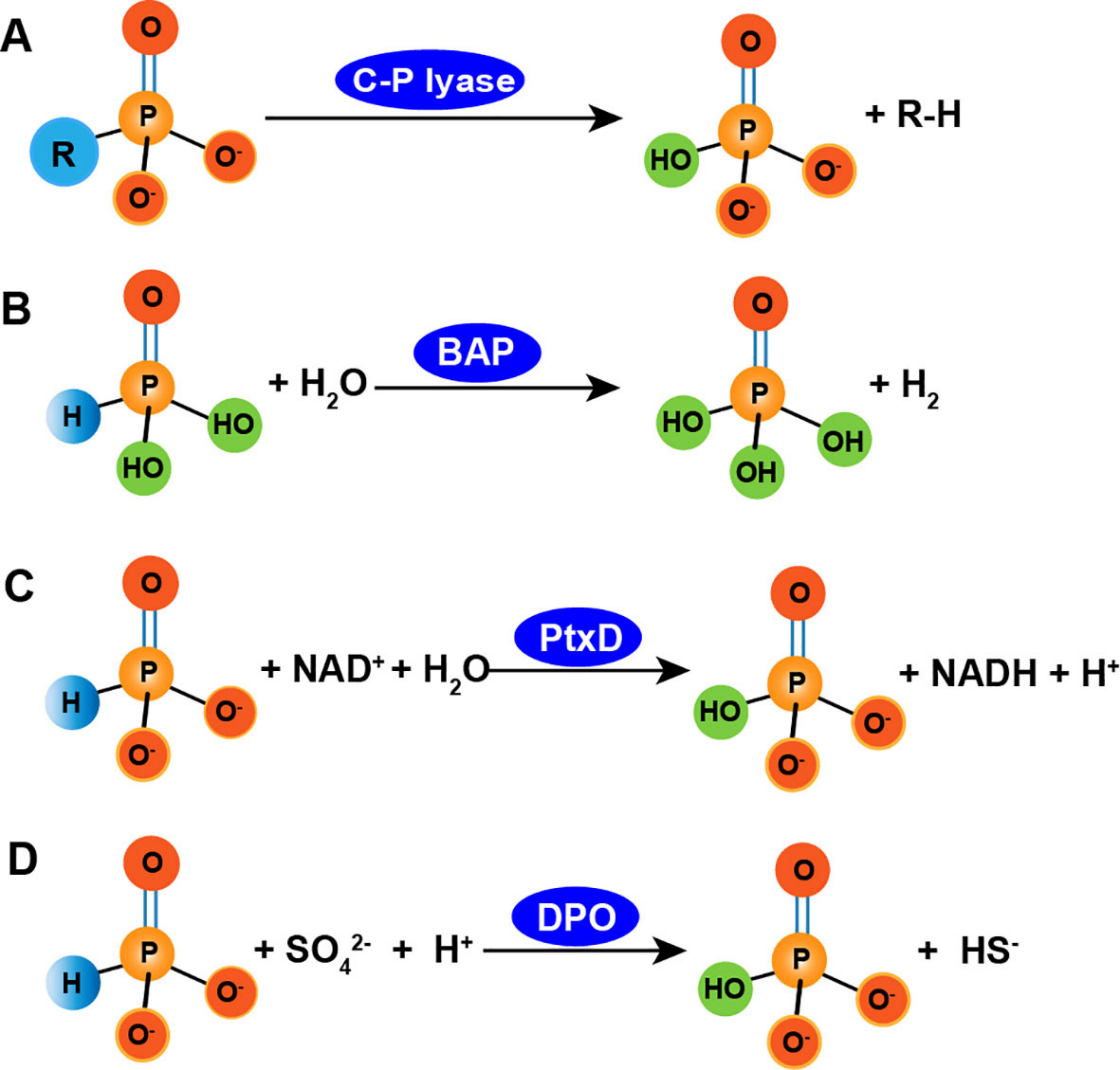
Several transformation routes of phosphite to phosphate. **A**, phosphite is degraded by C-P lyase (Seweryn et al., 2015); **B**, phosphite is oxidized by alkaline phosphatase such as phoA, with producing molecular H_2_ (Yang and Metcalf, 2004); **C**, phosphite is oxidized by ptxD enzyme, with producing NADH (Metcalf and Wolfe, 1998); **D**, phosphite is oxidized by dissimilatory phosphite oxidation such as *Desulfotignum phosphitoxidans* FiPS-3, *Candidatus Phosphitivorax* anaerolimi strain Phox-21 and Phosphitivorax strain Ca. P. anaerolimi F81, with simultaneously reducing sulphate to hydrogen sulphide (Helder et al., 2000).

Phi is oxidized into Pi by APO microorganisms and subsequently metabolized by microbial cells (Ewens et al., 2021; Metcalf and Wolfe, 1998; White and Metcalf, 2004). To date, more than 20 microorganisms, including proteobacteria, firmicutes, and cyanobacteria, have been isolated and shown to possess APO capability under laboratory conditions (Liu et al., 2023). Genetic and biochemical studies of some of these organisms have revealed several enzymes capable of oxidizing Phi. Currently, three APO enzymes have been characterized: C-P lyase, alkaline phosphatase (BAP), and phosphite dehydrogenase (ptxD). C-P lyases, found in *Escherichia coli*, are able to metabolize Phi into phosphonates (Seweryn et al., 2015), although the exact reaction mechanism remains unknown. Inferencing the known mechanism by which methylphosphonate is degraded by C-P lyase, the oxidation of Phi to Pi may involve cleavage of free radical P-H bonds (**Figure 3**). Phi is also oxidized by the bacterial BAP enzyme in *E. coli*. BAP, a periplasmic protein encoded by the *phoA* gene (**Figure 3**), is involved in the hydrolysis of Pi for P acquisition during Pi starvation (Yang and Metcalf, 2004). In addition, BAP oxidizes Phi to Pi and produces a hydrogen molecule *in vitro* (Yang and Metcalf, 2004).

In 1998, Metcalf and Wolf discovered that *Pseudomonas stutzeri* WM88 oxidized Phi to Pi (Metcalf and Wolfe, 1998). Subsequently, Costas et al. (2001) identified phosphite dehydrogenase (PTDH) from *Pseudomonas stutzeri* WM88, which can oxidize Phi (Costas et al., 2001). The PTDH enzyme complex contains five genes, *ptxA*, *ptxB*, *ptxC*, *ptxD*, and *ptxE*, that are involved in hypophosphite metabolism and belong to the *ptx* gene family. The proteins encoded by *ptxABC* are associated with the uptake and transport of Phi (Bisson et al., 2017), while *ptxD* encodes PTDH and while *ptxE* encodes a transcriptional regulatory factor (White and Metcalf, 2004). The PtxD enzyme oxidizes Phi *in vitro* using oxidized nicotinamide adenine dinucleotide (NAD^+^) as the only cofactor, producing Pi and reduced NAD (NADH) (**Figure 3**) (White and Metcalf, 2004). It only uses NAD^+^ as its coenzyme for catalysis and exhibits an extremely low affinity for NADP^+^ (White and Metcalf, 2004). The reaction catalyzed by PTDH proceeds in a continuous and orderly manner: PTDH first binds to NAD^+^, undergoes a conformational change, and forms a pocket-like structure that binds to the Phi substrate. The product, orthophosphate, is released first, followed by NADH once the catalytic reaction is complete.

In the DPO process, Phi serves as an electron donor and energy source for microbial growth and carbon fixation (Helder et al., 2000). DPO microorganisms include *Desulfotignum phosphitoxidans* FiPS-3, *Candidatus Phosphitivorax* anaerolimi strain Phox-21 (Figueroa and Coates, 2017; Schink et al., 2002), and the *Phosphitivorax* strain Ca. P. anaerolimi F81, which is isolated from Danish wastewater (Figueroa et al., 2017). *D. Phosphitoxidans* strain FiPS-3 is a newly discovered sulfate-reducing bacterium isolated from marine sediments. It has a slow propagation rate, doubling its population in 72 to 80 hours. It uses Phi as the sole electron donor and CO_2_ as the only carbon source. It can oxidize substrates, such as Phi, fumarate, pyruvate, glycine, glutamate, and maleate while reducing sulfate to sulfide (Poehlein et al., 2013; Schink et al., 2002). The five genes associated with *D. Phosphitoxidans* (*ptdFCGHI*) have only been identified in strain FiPS-3. PtdC is an inner membrane transporter facilitates Phi uptake, likely functioning as a Phi/Pi antiporter. PtdFGHI is potentially involved in energy conservation during DPO, but its function has not been experimentally confirmed (Figueroa et al., 2017; Figueroa and Coates, 2017; Poehlein et al., 2013).

### 4.2 Transformation of the *ptxD* gene in plants

Accumulation of Phi in plants significantly inhibits growth and can lead to death by interfering with the signaling pathways of PSR (Singh et al., 2003). The discovery of PTDH in microorganisms has enabled the use of Phi as an effective Phi-based fertilizer and herbicide (Achary et al., 2017; Heuer et al., 2017). The development of the *ptxD*/Phi system offers an efficient solution to the current challenges of P availability in soil and the evolution of herbicide-resistant weeds (**Table 2**). Phi has been used as a broad-spectrum and non-selective herbicide. It also improves crop productivity (Achary et al., 2017) and reduces Pi input by 30% to 50% (Herrera-Estrella and Lopez-Arredondo, 2016).

To our knowledge, Lopez-Arredondo and Herrera-Estrella (2012) overexpressed the *ptxD* gene in *Arabidopsis* and tobacco (*Nicotiana tabacum*), enabling the transgenic plants to oxidize Phi to Pi. Phi provides P nutrition, thereby reducing Pi fertilizer use by 30%–50% while effectively inhibiting the growth of weeds, such as *Brachypodium distachyon*, Alexander grass (*Brachiaria plantaginea*), morning-glory (*Ipomoea purpurea*), and smooth pigweed (*Amaranthus hybridus*). Foliar application of Phi can effectively inhibit or even eradicate broad-leaved weeds (Lopez-Arredondo and Herrera-Estrella, 2012). The transgenic rice lines harboring the *ptxD* gene exhibited vigorous growth and normal development of root systems. Additionally, both the aboveground and underground biomass of these transgenic plants were significantly higher than those of the wild-type counterparts (Manna et al., 2016). Nahampun et al. (2016) introduced a codon-optimized *ptxD* gene into corn. The callus tissue of the transgenic corn could grow normally on the medium with Phi as the P source (Nahampun et al., 2016). Similarly, Pandeya et al. (2018) overexpressed the *ptxD* gene in cotton (*Gossypium hirsutum*), which grew normally with Phi as the sole P source (Pandeya et al., 2018). The transgenic cotton plants accumulated 330%–480% more biomass than those grown under Pi conditions when cultivated with 80 mg/kg or 120 mg/kg Phi. Phi also significantly inhibited the growth of the glyphosate-resistant weed Palmer amaranth (*Amaranthus palmeri*) (**Table 2**) (Pandeya et al., 2018). Xu et al. (2024) transformed *ptxD* into rapeseed (*Brassica napus*) (**Table 2**). Notably, the transgenic rapeseed grew normally, while weeds were significantly inhibited when subjected to foliar fertilization with 200 mM K_2_HPO_4_ (Xu et al., 2024) (**Table 2**).

Hirota et al. (2012) isolated a soluble and heat-resistant Phi dehydrogenase, PTDH-R, from *Ralstonia* sp. strain 4506, which exhibited a higher affinity for Phi and a catalytic efficiency nearly six times that of PTDH-P (Hirota et al., 2012). The stability of the two catalytic domains may increase when the 139^th^ amino acid of PTDH mutates from tyrosine to glutamine or phenylalanine, enhancing PtxD activity (Liu et al., 2021). Overexpression of the mutated *ptxD_Q_* in *Arabidopsis* and rice effectively promoted plant growth when Phi was the sole P source (Liu et al., 2021). Enrique Asin-Garcia et al. (2022) integrated the Phi assimilation gene into the *Pseudomonas putida* KT2440 genome and knocked out the orthophosphate transporter gene to produce the PSAG-9 strain (Asin-Garcia et al., 2022). Notably, PSAG-9 acquired the ability to utilize *Pseudomonas putida* and could be cultured with Phi as the sole P source under non-sterile conditions (Asin-Garcia et al., 2022). These findings highlight the potential for Phi industrial application and release into the environment.

In 2004, Yang and Metcalf identified a BAP enzyme from *E. coli phn* mutants that was involved in Phi oxidation (Yang and Metcalf, 2004). Ram et al. (2019) transformed the codon-optimized *phoA* gene of *Desulfotignum phosphitoxidans* into rice. The results showed that transgenic rice grew healthily under 15 mM Phi, and the seeds germinated normally under 10 mM Phi (Ram et al., 2019). In addition to the transgenic rice, weeds such as *Phyllanthus urinaria*, *Portulaca oleracea* and *Amaranthus* sp. were arrested when sprayed with 100 mM potassium Phi (Ram et al., 2019) (**Table 2**).

**Table 2:** Overexpressed *ptxd* or *phoA* in different plants for weed control.

Plant	Gene	phosphite functions in weed control	Reference
*Arabidopsis thaliana* and tobacco	*ptxD*	80 mg/kg-120 mg/kg for soil fertilization 100 mM KH_2_PO_3_ for foliar fertilization	(Lopez-Arredondo and Herrera-Estrella, 2012)
tobacco	*ptxD* *phoA*	100 mM KH_2_PO_3_ 120mm Na_2_HPO_3_	(Lopez-Arredondo and Herrera-Estrella, 2012) (Yuan et al., 2021)
rice	*ptxD* *phoA*	500 mM Na_2_HPO_3_ 100 mM potassium phosphite	(Manna et al., 2016) (Ram et al., 2019)
maize transformation	*ptxD*	1.25–5 mM KH_2_PO_3_	(Nahampun et al., 2016)
cotton	*ptxD*	80 mg/kg or 120 mg/kg K_2_HPO_3_	(Pandeya et al., 2018)
rapeseed	*ptxD*	200 mg/L K_2_HPO_3_	(Xu et al., 2024)

The plants and its Latin names are as follows: tobacco (*Nicotiana tabacum*), rice (*Oryza sativa*), maize (*Zea mays*), cotton (*Gossypium hirsutum*) and rapeseed (*Brassica napus*).

Traditional herbicides target specific enzymes by binding to their catalytic sites of the enzyme (Heap and Duke, 2018). However, a few mutations in the active site can significantly reduce the binding of the herbicide to its target site, leading to the rapid evolution of herbicide-resistant weeds. In contrast, the utilization of Phi significantly reduces the probability of plant mutations because the development of point mutations requires the replacement of amino acids in multiple target proteins within the cell, which is highly unlikely to occur. Dominant mutations in multiple targets in the cell are lethal to the entire plant and are, therefore, completely unfeasible (Achary et al., 2017). Moreover, Phi can be degraded by soil microorganisms with no residual effects on subsequent crop rotations, which benefits both the environment and human health (Achary et al., 2017). Therefore, the dual fertilization and weed control system, which allows plants to use Phi as the sole P source, can potentially slow the evolution of herbicide-resistant weeds. To date, no genetically modified crops expressing recombinant *ptxD* or *BAP* have been commercially released worldwide.

### 4.3 Application of transgenic algae containing the *ptxD* gene

Phi-based fertilizers may reduce the occurrence of algae because Phi does not promote algal reproduction and has no toxic effects on algae due to their inability to metabolize Phi (Achary et al., 2017; Loera-Quezada et al., 2015, 2016). In the long term, Phi does not pose a threat to the species diversity of algae in aquatic ecosystems and can help maintain the equilibrium of the ecosystem (Loera-Quezada et al., 2015). Loera-Quezada et al. (2016) demonstrated that the introduction of *ptxD* into the nucleus of *Chlamydomonas reinhardtii* yielded transgenic lines capable of growing in media containing Phi as the sole P source, even under non-sterile conditions. These strains have a significant selective advantage over contaminating or competing species (Loera-Quezada et al., 2016). Changko et al. (2020) expressed the *ptxD* gene in the chloroplasts of *Chlamydomonas reinhardtii*, enabling it to grow in Phi-based media without any negative effects on its growth rate. However, the growth of *Chlamydomonas reinhardtii* in Phi media was severely hindered under contaminated microbial conditions (Changko et al., 2020).

Cutolo et al. (2020) transferred a mutated version of the *ptxD* gene into *Chlamydomonas reinhardtii* and used a mixture of NADP ^+^ and NAD^+^ to efficiently convert Phi into Pi, making this system an environmentally friendly alternative to antibiotic resistance genes for large-scale cultivation and application (Cutolo et al., 2020). Enrique Asin-Garcia et al. (2022) integrated the Phi assimilation gene into the *Pseudomonas putida* KT2440 genome and knocked out the orthophosphate transporter gene to produce the PSAG-9 strain (Asin-Garcia et al., 2022). PSAG-9 acquired the ability of *Pseudomonas putida* and could be cultured with Phi as the sole P source under non-sterile conditions (Asin-Garcia et al., 2022). These findings highlight the potential industrial applications of Phi and its release into the environment. Phi effectively inhibits the large-scale reproduction of green algae when used as a Pi fertilizer. Using Phi as a Pi fertilizer is important for protecting water bodies from P pollution because algae metabolize and utilize Phi, which is nearly non-toxic to algae and other aquatic organisms.

## 5 Development of Phi selectable marker in transgenic plants

Currently, antibiotic and herbicide-resistance genes are the most commonly used selectable markers in transgenic plants (Dormatey et al., 2021). However, resistance marker genes present within the plant genome can be inherited by offspring after the screening process, raising concerns regarding the food and environmental safety of transgenic plants. The *ptxD* gene is an ideal selectable marker that only confers resistance to Phi (Changko et al., 2020; Dormatey et al., 2021; Kanda et al., 2014; Nahampun et al., 2016), as it converts Phi into Pi, which plants can utilize for enhanced plant growth. This approach eliminates the risk of false positive clones and the escape of selectable markers. In genetic engineering, Phi serves as a selectable agent, and *ptxD* acts as a positive selectable marker in tissue culture technology (Nahampun et al., 2016; Pandeya et al., 2017).

Transgenic tobacco seedlings were screened from co-cultured leaf disc explants using a nutrient medium containing Phi as the P source and selectable agent (Lopez-Arredondo and Herrera-Estrella, 2013). Pandeya et al. (2017) transferred the *PtxD* gene into cotton and developed an efficient and straightforward screening method for transgenic cotton plants with the *ptxD*/Phi system. The transformation rate with the *ptxD*/Phi selection system was 19.10% and 38.87% higher than that of the *nptII*/Kan and *hpt*/Hyg B selection systems, respectively (Pandeya et al., 2017). The positive transformation rate using the *ptxD*/Phi selection system was 3.43%, compared to only 0.41% for the *bar*/PPT selection system (Pandeya et al., 2017).

The *ptxD*/Phi selection system has been successfully applied in the *Agrobacterium*-mediated transformation of maize (*Zea mays*) callus culture. Notably, its transformation success rate was comparable to that of the herbicide *bar*/bialaphos system (Nahampun et al., 2016).The *ptxD* gene is also used as a dominant marker for the genetic engineering of *Chlamydomonas reinhardtii* strains, thereby avoiding the use of antibiotic resistance genes as markers and enabling further modifications to existing engineered strains (Changko et al., 2020). In addition, oil-producing green algae (*Picochlorum* spp.) utilize *ptxD* as a selectable marker, with strains containing the *ptxD* gene able to grow on media with Phi as the sole P source (Dahlin and Guarnieri, 2022). Theoretically, *ptxD* can be used as a selectable marker for chloroplast engineering in other microalgae and as an introduction marker for Phi metabolism in important industrial strains (Changko et al., 2020). Moreover, bacterial alkaline phosphatase (BAP) has also been shown to oxidize Phi to Pi. Therefore, the *BAP*/Phi system could potentially replace the PTDH/Phi selectable marker agent combination for genetic transformation in plants (Yuan et al., 2021).

## 6 Conclusions and prospects

Given the rapid depletion of P resources and growing environmental awareness, there is an increasing focus on developing new phosphate fertilizers. Phi’s unique chemical properties and biological characteristics make it versatile for use as a fertilizer, bio-stimulant, fungicide, herbicide, and selectable marker. Phi has been identified in various environments, and its properties, mechanisms of action, and applications in agricultural production have been extensively studied. As an environmentally friendly fungicide and insecticide, Phi reduces the reliance on chemical pesticides and helps prevent the development of resistance to pathogens. Phi is harmless to humans and the environment, making it particularly useful for protecting horticultural plants. In modern agricultural systems, the enhancement of crop quality can be achieved by applying an optimal concentration of Phi in combination with other metallotrophic ions. Phi also introduces better strategies to address various environmental challenges, such as water scarcity, heat, and UV radiation. While, chemical oxidation of Phi in soil occurs slowly, microbial oxidation processes, including DPO and APO, can lead to the production of available Pi. The discovery of the *ptxd* gene has enabled plants to utilize Phi, leading to the development of herbicides and high-efficiency phosphate fertilizers. In addition, it can prevent the emergence of super-weeds, reduce the use of antibiotics in genetic engineering, eliminate the risk of false positive clones, and help prevent agricultural eutrophication. With P reserves rapidly declining, Phi is expected to become a crucial substitute for Pi, driving the development of modern and sustainable agriculture.

Genomic advances have enabled the development of a new generation of precisely formulated fertilizers that address farmers’ challenges while also creating opportunities to uncover new mechanisms triggered by Phi and expand its applications. Future studies should focus on the following aspects. (1) The assimilation, transport, and metabolic mechanisms of Phi in different plants, as well as the molecular basis of its interaction with plant hormones. (2) Further investigation into the genes involved in the transformation of Phi to Pi in the DPO process of microorganism and their potential functions. (3) The development of genetically modified crops that efficiently utilize Phi through gene-editing and other genetic engineering tools. (4) The development of new Phi preparations to enhance their applicability and environmental friendliness in agricultural production. Notably, the residual presence of Phi in fruits is a major concern, and the potential threats to the environment (air, soil and water) and human health from large-scale usage should be carefully assessed.

## References

[B1] Abbasi P. A. Lazarovits G. (2006). Seed treatment with phosphonate (AG3) suppresses Pythium damping-off of cucumber seedlings. Plant Dis. 90, 459–464. doi: 10.1094/PD-90-0459 30786594

[B2] Achary V. M. M. Ram B. Manna M. Datta D. Bhatt A. Reddy M. K.. (2017). Phosphite: a novel P fertilizer for weed management and pathogen control. Plant Biotechnol. J. 15, 1493–1508. doi: 10.1111/pbi.12803 28776914 PMC5698055

[B3] Aćimović S. G. VanWoerkom A. H. Garavaglia T. Vandervoort C. Sundin G. W. Wise J. C. (2016). Seasonal and cross-seasonal timing of fungicide trunk injections in apple trees to optimize management of apple scab. Plant Dis. 100, 1606–1616. doi: 10.1094/pdis-09-15-1061-re 30686216

[B4] Andreu A. B. Guevara M. G. Wolski E. A. Daleo G. R. Caldiz D. O. (2006). Enhancement of natural disease resistance in potatoes by chemicals. Pest Manage. Sci. 62, 162–170. doi: 10.1002/ps.1142 16408317

[B5] Asin-Garcia E. Batianis C. Li Y. Fawcett J. D. de Jong I. dos Santos V. A. P. M. (2022). Phosphite synthetic auxotrophy as an effective biocontainment strategy for the industrial chassis Pseudomonas putida. Microb. Cell Fact. 21, 156. doi: 10.1186/s12934-022-01883-5 35934698 PMC9358898

[B6] Ávila F. W. Faquin V. Lobato A. Ávila P. A. Marques D. J. Guedes E. M. S.. (2013). Effect of phosphite supply in nutrient solution on yield, phosphorus nutrition and enzymatic behavior in common bean (*Phaseolus vulgaris* L.) plants. Aust. J. Crop Sci. 8, 67–74.

[B7] Bertsch F. Ramírez F. Henríquez C. (2009). Evaluación del fosfito como fuente fertilizante de fósforo vía radical y foliar. Agronomía Costarricense 33, 249–265. doi: 10.15517/rac.v33i2.6724

[B8] Bisson C. Adams N. B. P. Stevenson B. Brindley A. A. Polyviou D. Bibby T. S.. (2017). The molecular basis of phosphite and hypophosphite recognition by ABC-transporters. Nat. Commun. 8, 1746. doi: 10.1038/s41467-017-01226-8 29170493 PMC5700983

[B9] Bock C. H. Brenneman T. B. Hotchkiss M. W. Wood B. W. (2012). Evaluation of a phosphite fungicide to control pecan scab in the southeastern USA. Crop Prot. 36, 58–64. doi: 10.1016/j.cropro.2012.01.009

[B10] Borza T. Schofield A. Sakthivel G. Bergese J. Gao X. Rand J.. (2014). Ion chromatography analysis of phosphite uptake and translocation by potato plants: Dose-dependent uptake and inhibition of *Phytophthora infestans* development. Crop Prot. 56, 74–81. doi: 10.1016/j.cropro.2013.10.024

[B11] Burra D. D. Berkowitz O. Hedley P. E. Morris J. Resjö S. Levander F.. (2014). Phosphite-induced changes of the transcriptome and secretome in Solanum tuberosum leading to resistance against *Phytophthora infestans* . BMC Plant Biol. 14, 254. doi: 10.1186/s12870-014-0254-y 25270759 PMC4192290

[B12] Carmona M. A. Sautua F. J. Grijalba P. E. Cassina M. Perez-Hernandez O. (2018). Effect of potassium and manganese phosphites in the control of Pythium damping-off in soybean: a feasible alternative to fungicide seed treatments. Pest Manage. Sci. 74, 366–374. doi: 10.1002/ps.4714 28842951

[B13] Carswell C. Grant B. R. Theodorou M. E. Harris J. Niere J. O. Plaxton W. C. (1996). The fungicide phosphonate disrupts the phosphate-starvation response in *Brassica nigra* seedlings. Plant Physiol. 110, 105–110. doi: 10.1104/pp.110.1.105 12226174 PMC157699

[B14] Cerqueira A. Alves A. Berenguer H. Correia B. Gómez-Cadenas A. Diez J. J.. (2017). Phosphite shifts physiological and hormonal profile of Monterey pine and delays *Fusarium circinatum* progression. Plant Physiol. Biochem. 114, 88–99. doi: 10.1016/j.plaphy.2017.02.020 28284060

[B15] Changko S. Rajakumar P. D. Young R. E. B. Purton S. (2020). The phosphite oxidoreductase gene, ptxD as a bio-contained chloroplast marker and crop-protection tool for algal biotechnology using *Chlamydomonas* . Appl. Microbiol. Biotechnol. 104, 675–686. doi: 10.1007/s00253-019-10258-7 31788712 PMC6943410

[B16] Coffey M. Joseph M. (1985). Effects of phosphorous acid and fosetyl-Al on the life cycle of *Phytophthora cinnamomi* and *Phytophthora citricola* . Phytopathology 75, 1042–1046. doi: 10.1094/phyto-75-1042

[B17] Costas A. M. White A. K. Metcalf W. W. (2001). Purification and characterization of a novel phosphorus-oxidizing enzyme from *Pseudomonas stutzeri* WM88. J. Biol. Chem. 276, 17429–17436. doi: 10.1074/jbc.M011764200 11278981

[B18] Cutolo E. Tosoni M. Barera S. Herrera-Estrella L. Dall’Osto L. Bassi R. (2020). A phosphite dehydrogenase variant with promiscuous access to nicotinamide cofactor pools sustains fast phosphite-dependent growth of transplastomic *Chlamydomonas reinhardtii* . Plants 9, 473. doi: 10.3390/plants9040473 32276527 PMC7238262

[B19] Dahlin L. R. Guarnieri M. T. (2022). Heterologous expression of phosphite dehydrogenase in the chloroplast or nucleus enables phosphite utilization and genetic selection in Picochlorum spp. Algal Res. 62, 102604. doi: 10.1016/j.algal.2021.102604

[B20] Daniel R. Guest D. (2005). Defence responses induced by potassium phosphonate in Phytophthora palmivora-challenged *Arabidopsis thaliana* . Physiol. Mol. Plant Pathol. 67, 194–201. doi: 10.1016/j.pmpp.2006.01.003

[B21] Danova-Alt R. Dijkema C. W. P. D. E. Kock M. (2008). Transport and compartmentation of phosphite in higher plant cells–kinetic and P nuclear magnetic resonance studies. Plant Cell Environ. 31, 1510–1521. doi: 10.1111/j.1365-3040.2008.01861.x 18657056

[B22] Dias-Arieira C. R. Marini P. M. Fontana L. F. Roldi M. Silva T. (2012). Effect of azospirillum brasilense, stimulate and potassium phosphite to control pratylenchus brachyurus in soybean and maize. Nematropica 42, 170–175.

[B23] Dormatey R. (2022). Physiological and Molecular Analysis of Phosphite Response in Potato (*Solanum tuberosum* L.) (Lanzhou, China: Doctoral Dissertation, Gansu agricultural university).

[B24] Dormatey R. Sun C. Ali K. Fiaz S. Xu D. Calderón-Urrea A.. (2021). ptxD/Phi as alternative selectable marker system for genetic transformation for bio-safety concerns: a review. PeerJ 9, e11809. doi: 10.7717/peerj.11809 34395075 PMC8323600

[B25] Eshraghi L. Anderson J. Aryamanesh N. Shearer B. McComb J. Hardy G. E. S.. (2011). Phosphite primed defence responses and enhanced expression of defence genes in *Arabidopsis thaliana* infected with *Phytophthora cinnamomi* . Plant Pathol. 60, 1086–1095. doi: 10.1111/j.1365-3059.2011.02471.x

[B26] Estrada-Ortiz E. Trejo-Téllez L. I. Gómez-Merino F. C. Núñez-Escobar R. Sandoval-Villa M. (2013). The effects of phosphite on strawberry yield and fruit quality. J. Soil Sci. Plant Nut 13, 612–620. doi: 10.4067/S0718-95162013005000049

[B27] Estrada-Ortiz E. Trejo-Téllez L. I. Gómez-Merino F. C. Silva-Rojas H. V. Castillo-González A. M. Avitia-García E. (2016). Physiological responses of chard and lettuce to phosphite supply in nutrient solution. J. Agr. Sci. Tech. 18, 1079–1090.

[B28] Ewens S. D. Gomberg A. F. S. Barnum T. P. Borton M. A. Carlson H. K. Wrighton K. C.. (2021). The diversity and evolution of microbial dissimilatory phosphite oxidation. Proc. Natl. Acad. Sci. U.S.A. 118, e2020024118. doi: 10.1073/pnas.2020024118 33688048 PMC7980464

[B29] Fagundes-Nacarath I. R. F. Debona D. Oliveira A. T. H. Hawerroth C. Rodrigues F. A. (2018). Biochemical responses of common bean to white mold potentiated by phosphites. Plant Physiol. Biochem. 132, 308–319. doi: 10.1016/j.plaphy.2018.09.016 30248517

[B30] Figueira E. P. P. Kuhn O. J. Martinazzo-Portz T. Stangarlin J. R. Pereira M. D. P. Lampugnani C. (2020). Histochemical changes induced by Trichoderma spp. and potassium phosphite in common bean (*Phaseolus vulgaris*) in response to the attack by *Colletotrichum lindemuthianum* . Semin. Ciênc. Agrár 41, 811–828. doi: 10.5433/1679-0359.2020v41n3p811

[B31] Figueroa I. A. Barnum T. P. Somasekhar P. Y. Carlström C. I. Engelbrektson A. L. Coates J. D. (2017). Metagenomics-guided analysis of microbial chemolithoautotrophic phosphite oxidation yields evidence of a seventh natural CO^2^ fixation pathway. Proc. Natl. Acad. Sci. U.S.A. 115 (1), E92–E101. doi: 10.1073/pnas.1715549114 29183985 PMC5776814

[B32] Figueroa I. A. Coates J. D. (2017). Microbial phosphite oxidation and its potential role in the global phosphorus and carbon cycles. Adv. Appl. Microbiol. 98, 93–117. doi: 10.1016/bs.aambs.2016.09.004 28189156

[B33] Glinicki R. Sas-Paszt L. Jadczuk-Tobjasz E. (2010). The effect of plant stimulant fertilizer resistim on growth and development of strawberry plants. J. Fruit Ornam. Plant Res. 18, 111–124.

[B34] Gómez-Merino F. C. Gómez-Trejo L. F. Ruvalcaba-Ramírez R. Trejo-Téllez L. I. (2022). Chapter 7 – application of phosphite as a biostimulant in agriculture. New Fut. Dev. Microbial Biotechnol. Bioengin. 135–153.

[B35] Gómez-Merino F. C. Trejo-Téllez L. I. (2015). Biostimulant activity of phosphite in horticulture. Sci. Hortic. 196, 82–90. doi: 10.1016/j.scienta.2015.09.035

[B36] Grant B. R. Grant J. H. Harris J. (1992). Inhibition of growth of *Phytophthora infestans* by phosphate and phosphonate in defined media. Exp. Mycol. 16, 240–244. doi: 10.1016/0147-5975(92)90032-M

[B37] Griffith J. M. Akins L. A. Grant B. R. (1989). Properties of the phosphate and phosphite transport systems of *Phytophthora palmivora* . Arch. Microbiol. 152, 430–436. doi: 10.1007/BF00446924

[B38] Guest D. Grant B. (1991). The complex action of phosphonates as antifungal agents. Biol. Rev. 66, 159–187. doi: 10.1111/j.1469-185X.1991.tb01139.x

[B39] Guo M. Li B. Xiang Q. Wang R. Liu P. Chen Q. (2021). Phosphite translocation in soybean and mechanisms of *Phytophthora sojae* inhibition. Pestic. Biochem. Phys. 172, 104757. doi: 10.1016/j.pestbp.2020.104757 33518050

[B40] Han C. Geng J. Ren H. Gao S. Xie X. Wang X. (2013). Phosphite in sedimentary interstitial water of Lake Taihu, a large eutrophic shallow lake in China. Environ. Sci. Technol. 47, 5679–5685. doi: 10.1021/es305297y 23647420

[B41] Han X. Xi Y. Zhang Z. Mohammadi M. A. Joshi J. Borza T.. (2021). Effects of phosphite as a plant biostimulant on metabolism and stress response for better plant performance in *Solanum tuberosum* . Ecotoxicol. Environ. Saf. 210, 111873. doi: 10.1016/j.ecoenv.2020.111873 33418157

[B42] Havlin J. L. Schlegel A. J. (2021). Review of phosphite as a plant nutrient and fungicide. Soil Syst. 5, 52. doi: 10.3390/soilsystems5030052

[B43] Heap I. Duke S. O. (2018). Overview of glyphosate-resistant weeds worldwide. Pest Manage. Sci. 74, 1040–1049. doi: 10.1002/ps.4760 29024306

[B44] Helder J. Schots A. Bakker J. Smant G. (2000). Bacterial metabolism e phosphite oxidation by sulphate reduction. Nature 46, 37. doi: 10.1038/35017644 10894531

[B45] Herrera-Estrella L. Lopez-Arredondo D. (2016). Phosphorus: the underrated element for feeding the world. Trends Plant Sci. 21, 461–463. doi: 10.1016/j.tplants.2016.04.010 27160806

[B46] Heuer S. Gaxiola R. Schilling R. Herrera-Estrella L. Lopez-Arredondo D. Wissuwa M.. (2017). Improving phosphorus use efficiency: a complex trait with emerging opportunities. Plant J. 90, 868–885. doi: 10.1111/tpj.13423 27859875

[B47] Hirosse E. H. Creste J. E. Custodio C. C. MaChado-Neto N. B. (2012). *In vitro* growth of sweet potato fed with potassium phosphite. Acta Sci. Agron. 34, 85–91. doi: 10.4025/actasciagron.v34i1.10810

[B48] Hirota R. Yamane S.-t. Fujibuchi T. Motomura K. Ishida T. Ikeda T.. (2012). Isolation and characterization of a soluble and thermostable phosphite dehydrogenase from Ralstonia sp. strain 4506. J. Biosci. Bioeng. 113, 445–450. doi: 10.1016/j.jbiosc.2011.11.027 22197497

[B49] Hofgaard I. S. Ergon A. Henriksen B. Tronsmo A. M. (2010). The effect of potential resistance inducers on development of *Microdochium majus* and *Fusarium culmorum* in winter wheat. Eur. J. Plant Pathol. 128, 269–281. doi: 10.1007/s10658-010-9662-5

[B50] Huang Y. Cai S. Zhang G. Ruan S. (2020). Transcriptome-based analysis of phosphite-induced resistance against pathogens in rice. Plants (Basel) 9, 1334. doi: 10.3390/plants9101334 33050314 PMC7650589

[B51] Hunter S. McDougal R. Williams N. Scott P. (2023). Evidence of phosphite tolerance in *Phytophthora cinnamomi* from New Zealand avocado orchards. Plant Dis. 107, 393–400. doi: 10.1094/pdis-05-22-1269-re 36089692

[B52] Jost R. Pharmawati M. Lapis-Gaza H. R. Rossig C. Berkowitz O. Lambers H.. (2015). Differentiating phosphate-dependent and phosphate-independent systemic phosphate-starvation response networks in *Arabidopsis thaliana* through the application of phosphite. J. Exp. Bot. 66, 2501–2514. doi: 10.1093/jxb/erv025 25697796 PMC4986860

[B53] Kanda K. Ishida T. Hirota R. Ono S. Motomura K. Ikeda T.. (2014). Application of a phosphite dehydrogenase gene as a novel dominant selection marker for yeasts. J. Biotechnol. 182–183, 68–73. doi: 10.1016/j.jbiotec.2014.04.012 24786825

[B54] Kehler A. Haygarth P. Tamburini F. Blackwell M. (2021). Cycling of reduced phosphorus compounds in soil and potential impacts of climate change. Eur. J. Soil Sci. 72, 2517–2537. doi: 10.1111/ejss.13121

[B55] Li Z. Wu Y. Hu J. Yang G. Wang Z. Sun J. (2022). Dissection of the response mechanism of alfalfa under phosphite stress based on metabolomic and transcriptomic data. Plant Physiol. Biochem. 192, 35–49. doi: 10.1016/j.plaphy.2022.09.024 36206705

[B56] Liljeroth E. Lankinen Å. Wiik L. Burra D. D. Alexandersson E. Andreasson E. (2016). Potassium phosphite combined with reduced doses of fungicides provides efficient protection against potato late blight in large-scale field trials. Crop Prot. 86, 42–55. doi: 10.1016/j.cropro.2016.04.003

[B57] Liu T. Yuan L. Deng S. Zhang X. Cai H. Ding G.. (2021). Improved the activity of phosphite dehydrogenase and its application in plant biotechnology. Front. Bioeng. Biotechnol. 9. doi: 10.3389/fbioe.2021.764188 PMC8655118 34900961

[B58] Liu W. Zhang Y. Yu M. Xu J. Du H. Zhang R.. (2023). Role of phosphite in the environmental phosphorus cycle. Sci. Total. Environ. 881, 163463. doi: 10.1016/j.scitotenv.2023.163463 37062315

[B59] Lobato M. C. MaChinandiarena M. F. Tambascio C. Dosio G. A. A. Caldiz D. O. Daleo G. R.. (2011). Effect of foliar applications of phosphite on post-harvest potato tubers. Eur. J. Plant Pathol. 130, 155–163. doi: 10.1007/s10658-011-9741-2

[B60] Lobato M. C. Olivieri F. P. Daleo G. R. Andreu A. B. (2010). Antimicrobial activity of phosphites against different potato pathogens. J. Plant Dis. Protect. 117, 102–109. doi: 10.1007/BF03356343

[B61] Loera-Quezada M. M. Leyva-Gonzalez M. A. Lopez-Arredondo D. Herrera-Estrella L. (2015). Phosphite cannot be used as a phosphorus source but is non-toxic for microalgae. Plant Sci. 231, 124–130. doi: 10.1016/j.plantsci.2014.11.015 25575997

[B62] Loera-Quezada M. M. Leyva-Gonzalez M. A. Velazquez-Juarez G. Sanchez-Calderon L. Do Nascimento M. Lopez-Arredondo D.. (2016). A novel genetic engineering platform for the effective management of biological contaminants for the production of microalgae. Plant Biotechnol. J. 14, 2066–2076. doi: 10.1111/pbi.12564 27007496 PMC5043480

[B63] Lopez-Arredondo D. L. Herrera-Estrella L. (2012). Engineering phosphorus metabolism in plants to produce a dual fertilization and weed control system. Nat. Biotechnol. 30, 889–893. doi: 10.1038/nbt.2346 22922674

[B64] Lopez-Arredondo D. L. Herrera-Estrella L. (2013). A novel dominant selectable system for the selection of transgenic plants under *in vitro* and greenhouse conditions based on phosphite metabolism. Plant Biotechnol. J. 11, 516–525. doi: 10.1111/pbi.12063 23530523

[B65] Lovatt C. J. Mikkelsen R. L. (2006). Phosphite fertilizers? What are they? Can you use them? What can they do? Better Crops 90, 11–13.

[B66] MaChinandiarena M. F. Lobato M. C. Feldman M. L. Daleo G. R. Andreu A. B. (2012). Potassium phosphite primes defense responses in potato against *Phytophthora infestans* . J. Plant Physiol. 169, 1417–1424. doi: 10.1016/j.jplph.2012.05.005 22727804

[B67] Manna M. Achary V. M. Islam T. Agrawal P. K. Reddy M. K. (2016). The development of a phosphite-mediated fertilization and weed control system for rice. Sci. Rep. 6, 24941. doi: 10.1038/srep24941 27109389 PMC4842969

[B68] Manna M. Islam T. Kaul T. Reddy C. S. Fartyal D. James D.. (2015). A comparative study of effects of increasing concentrations of phosphate and phosphite on rice seedlings. Acta Physiol. Plant 37, 258. doi: 10.1007/s11738-015-2016-3

[B69] Marin M. V. Baggio J. S. Melo P. P. Peres N. A. (2023). Phosphite Is More Effective Against Phytophthora Crown Rot and Leather Rot Caused by *Phytophthora cactorum* than *P. nicotianae* . Plant Dis. 107, 1602–1608. doi: 10.1094/pdis-06-22-1481-re 36415890

[B70] Market Reports World Global Potassium Phosphite Market—Market Reports World. Available online at: https://www.marketreportsworld.com/global-potassium-phosphite-market-26318022 (Accessed 18 February 2024).

[B71] Martínez S. (2016). Effects of combined application of potassium phosphite and fungicide on stem and sheath disease control, yield, and quality of rice. Crop Prot. 89, 259–264. doi: 10.1016/j.cropro.2016.08.002

[B72] McDonald A. E. Grant B. R. Plaxton W. C. (2001a). Phosphite (phosphorous acid): its relevance in the environment and agriculture and influence on plant phosphate starvation response. J. Plant Nutr. 24, 1505–1519. doi: 10.1081/pln-100106017

[B73] McDonald A. E. Niere J. O. Plaxton W. C. (2001b). Phosphite disrupts the acclimation of *Saccharomyces cerevisiae* to phosphate starvation. Can. J. Microbiol. 47, 969–978. doi: 10.1139/w01-099 11766057

[B74] Metcalf W. W. Wolfe R. S. (1998). Molecular genetic analysis of phosphite and hypophosphite oxidation by *Pseudomonas stutzeri* WM88. J. Bacteriol. 180, 5547–5558. doi: 10.1128/jb.180.21.5547-5558.1998 9791102 PMC107611

[B75] Mohammadi M. A. Han X. Zhang Z. Xi Y. Boorboori M. Wang-Pruski G. (2020). Phosphite application alleviates *Pythophthora infestans* by modulation of photosynthetic and physio-biochemical metabolites in potato leaves. Pathogens 9, 170. doi: 10.3390/pathogens9030170 32121090 PMC7157663

[B76] Monsalve J. V. Viteri S. Viteri S. Cárdenas N. J. R. Duarte F. O. T. (2012). Effect of the Potasium Phosphite in Combination with the Fungicide Metalaxyl plus Mancozeb on the Control of Downy Mildew (*Peronospora destructor Berk*) in Onion Bulb (*Allium cepa* L.). Rev. Fac. Nac. Agron. Medellín 65, 6317–6325.

[B77] Moor U. Põldma P. Tõnutare T. Karp K. Starast M. Vool E. (2009). Effect of phosphite fertilization on growth, yield and fruit composition of strawberries. Sci. Hortic. 119, 264–269. doi: 10.1016/j.scienta.2008.08.005

[B78] Mooy B. A. S. V. Krupke A. Dyhrman S. T. Fredricks H. F. Frischkorn K. R. Ossolinski J. E.. (2015). Major role of planktonic phosphate reduction in the marine phosphorus redox cycle. Science 348, 783–785. doi: 10.1126/science.aaa8181 25977548

[B79] Nahampun H. N. Lopez-Arredondo D. Xu X. Herrera-Estrella L. Wang K. (2016). Assessment of ptxD gene as an alternative selectable marker for *Agrobacterium*-mediated maize transformation. Plant Cell Rep. 35, 1121–1132. doi: 10.1007/s00299-016-1942-x 26883223

[B80] Oka Y. Tkachi N. Mor M. (2007). Phytopathology-Phosphite Inhibits Development of the Nematodes *Heterodera avenae* and *Meloidogyne marylandi* in Cereals. Phytopathology 97, 396–404. doi: 10.1094/PHYTO-97-4-0396 18943279

[B81] Omar M. M. Taha A. A. Shokir S. (2020). Effect of applying potassium phosphite with potassium fulvate on plant growth. J. Soil Sci. Agr Eng. 11, 255–263. doi: 10.21608/jssae.2020.109423

[B82] Ouimette D. G. Coffey M. D. (1990). Symplastic entry and phloem translocation of phosphonate. Pestic. Biochem. Phys. 38, 18–25. doi: 10.1016/0048-3575(90)90143-P

[B83] Pandeya D. Campbell L. M. Nunes E. Lopez-Arredondo D. L. Janga M. R. Herrera-Estrella L.. (2017). ptxD gene in combination with phosphite serves as a highly effective selection system to generate transgenic cotton (*Gossypium hirsutum* L.). Plant Mol. Biol. 95, 567–577. doi: 10.1007/s11103-017-0670-0 29032395

[B84] Pandeya D. Lopez-Arredondo D. L. Janga M. R. Campbell L. M. Estrella-Hernandez P. Bagavathiannan M. V.. (2018). Selective fertilization with phosphite allows unhindered growth of cotton plants expressing the *ptxD* gene while suppressing weeds. Proc. Natl. Acad. Sci. U.S.A. 115, E6946–E6955. doi: 10.1073/pnas.1804862115 29866830 PMC6055188

[B85] Panicker S. Gangadharan K. (1999). Controlling downy mildew of maize caused by *Peronosclerospora sorghi* by foliar sprays of phosphoric acid compounds. Crop Prot. 18, 115–118. doi: 10.1016/S0261-2194(98)00101-X

[B86] Pasek M. A. Sampson J. M. Atlas Z. (2014). Redox chemistry in the phosphorus biogeochemical cycle. Proc. Natl. Acad. Sci. U.S.A. 111, 15468–15473. doi: 10.1073/pnas.1408134111 25313061 PMC4217446

[B87] Pérez-Zavala F. G. Ojeda-Rivera J. O. Herrera-Estrella L. López-Arredondo D. (2024). Beneficial effects of phosphite in *Arabidopsis thaliana* mediated by activation of ABA, SA, and JA biosynthesis and signaling pathways. Plants 13 (13), 1873. doi: 10.3390/plants13131873 38999712 PMC11244317

[B88] Poehlein A. Daniel R. Schink B. Simeonova D. D. (2013). Life based on phosphite_ a genome-guided analysis of *Desulfotignum phosphitoxidans* . BMC Genomics 14, 753. doi: 10.1186/1471-2164-14-753 24180241 PMC4046663

[B89] Pratt J. Boisson A. M. Gout E. Bligny R. Douce R. Aubert S. (2009). Phosphate (Pi) starvation effect on the cytosolic Pi concentration and Pi exchanges across the tonoplast in plant cells: an *in vivo* ^31^P-nuclear magnetic resonance study using methylphosphonate as a Pi analog. Plant Physiol. 151, 1646–1657. doi: 10.1104/pp.109.144626 19755536 PMC2773096

[B90] Ram B. Fartyal D. Sheri V. Varakumar P. Borphukan B. James D.. (2019). Characterization of phoA, a bacterial alkaline phosphatase for phi use efficiency in rice plant. Front. Plant Sci. 10. doi: 10.3389/fpls.2019.00037 PMC6397861 30858852

[B91] Rickard D. A. (2000). Review of phosphorus acid and its salts as fertilizer materials. J. Plant Nutr. 23, 161–180. doi: 10.1080/01904160009382006

[B92] Routray W. Orsat V. (2011). Blueberries and their anthocyanins: factors affecting biosynthesis and properties. Compr. Rev. Food Sci. F. 10, 303–320. doi: 10.1111/j.1541-4337.2011.00164.x

[B93] Schink B. Thiemann V. Laue H. Friedrich M. (2002). Desulfotignum phosphitoxidans sp. nov., a new marine sulfate reducer that oxidizes phosphite to phosphate. Arch. Microbiol. 177, 381–391. doi: 10.1007/s00203-002-0402-x 11976747

[B94] Seweryn P. Van L. B. Kjeldgaard M. Russo C. J. Passmore L. A. Hove-Jensen B.. (2015). Structural insights into the bacterial carbon–phosphorus lyase machinery. Nature 525, 68–72. doi: 10.1038/nature14683 26280334 PMC4617613

[B95] Silva O. C. Santos H. A. A. Dalla Pria M. May-De-Mio L. L. (2011). Potassium phosphite for control of downy mildew of soybean. Crop Prot. 30, 598–604. doi: 10.1016/j.cropro.2011.02.015

[B96] Singh V. K. Wood S. M. Knowles V. L. Plaxton W. C. (2003). Phosphite accelerates programmed cell death in phosphate-starved oilseed rape (*Brassica napus*) suspension cell cultures. Planta 218, 233–239. doi: 10.1007/s00425-003-1088-2 12920596

[B97] Smillie R. Grant B. Guest D. (1989). The mode of action of phosphite: evidence for both direct and indirect modes of action on three phytophthora sp. in plants. Phytopathology 79, 921–926. doi: 10.1094/PHYTO-79-921

[B98] Soledad O. N. Florencia M. M. Laura F. M. Raul D. G. Balbina A. A. Pia O. F. (2015). Potassium phosphite increases tolerance to UV-B in potato. Plant Physiol. Biochem. 88, 1–8. doi: 10.1016/j.plaphy.2015.01.003 25596554

[B99] Speiser B. Berner A. Häseli A. Tamm L. (2000). Control of downy mildew of grapevine with potassium phosphonate: effectivity and phosphonate residues in wine. Biol. Agric. Hortic. 17, 305–312. doi: 10.1080/01448765.2000.9754851

[B100] Su L. Qiu P. Fang Z. Sun J. Mo X. Liu Y.. (2022). Potassium phosphite enhances the antagonistic capability of *Bacillus amyloliquefaciens* to manage tomato bacterial wilt. Plant Dis. 106, 654–660. doi: 10.1094/PDIS-08-21-1601-RE 34491099

[B101] Tambascio C. Covacevich F. Lobato M. C. Lasa C.d. Caldiz D. Dosio G.. (2014). The application of K phosphites to seed tubers enhanced emergence, early growth and mycorrhizal colonization in potato (*American journal of plant sciences*). Am. J. Plant Sci. 05, 132–137. doi: 10.4236/ajps.2014.51017

[B102] Tapia-Torres Y. Rodríguez-Torres M. D. Elser J. J. Islas A. Souza V. García-Oliva F.. (2016). How to live with phosphorus scarcity in soil and sediment: lessons from bacteria. Appl. Environ. Microbiol. 82, 4652–4662. doi: 10.1128/aem.00160-16 27235437 PMC4984279

[B103] Thao H. T. B. Yamakawa T. (2009). Phosphite (phosphorous acid): Fungicide, fertilizer or bio-stimulator? Soil Sci. Plant Nutr. 55, 228–234. doi: 10.1111/j.1747-0765.2009.00365.x

[B104] Thao H. T. B. Yamakawa T. Myint A. K. Sarr P. S. (2008a). Effects of phosphite, a reduced form of phosphate, on the growth and phosphorus nutrition of spinach (*Spinacia oleracea* L.). Soil Sci. Plant Nutr. 54, 761–768. doi: 10.1111/j.1747-0765.2008.00290.x

[B105] Thao H. T. B. Yamakawa T. Shibata K. Sarr P. S. Myint A. K. (2008b). Growth response of komatsuna (*Brassica rapa* var. Peruviridis) to root and foliar applications of phosphite. Plant Soil 308, 1–10. doi: 10.1007/s11104-008-9598-0

[B106] Ticconi C. A. Delatorre C. A. Abel S. (2001). Attenuation of phosphate starvation responses by phosphite in *Arabidopsis* . Plant Physiol. 127, 963–972. doi: 10.1104/pp.010396 11706178 PMC129267

[B107] Trejo-Téllez L. I. Gómez-Merino F. C. (2018). “Phosphite as an inductor of adaptive responses to stress and stimulator of better plant performance,” Biotic and Abiotic Stress Tolerance in Plants. ed. Vats S. (Berlin: Springer), 203–238.

[B108] Varadarajan D. K. Karthikeyan A. S. Matilda P. D. Raghothama K. G. (2002). Phosphite, an analog of phosphate, suppresses the coordinated expression of genes under phosphate starvation. Plant Physiol. 129, 1232–1240. doi: 10.1104/pp.010835 12114577 PMC166517

[B109] Vinas M. Mendez J. C. Jiménez V. M. (2020). Effect of foliar applications of phosphites on growth, nutritional status and defense responses in tomato plants. Sci. Hortic. 265, 109200. doi: 10.1016/j.scienta.2020.109200

[B110] White A. K. Metcalf W. W. (2004). The *htx* and *ptx* operons of *Pseudomonas stutzeri* WM88 are new members of the pho regulon. J. Bacteriol. 186, 5876–5882. doi: 10.1128/JB.186.17.5876-5882.2004 15317793 PMC516845

[B111] White A. K. Metcalf W. W. (2007). Microbial metabolism of reduced phosphorus compounds. Annu. Rev. Microbiol. 61, 379–400. doi: 10.1146/annurev.micro.61.080706.093357 18035609

[B112] Wilkinson C. J. Holmes J. M. Dell B. Tynan K. M. McComb J. A. Shearer B. L.. (2001). Effect of phosphite on in planta zoospore production of *Phytophthora cinnamomi* . Plant Pathol. 50, 587–593. doi: 10.1046/j.1365-3059.2001.00605.x

[B113] Xi Y. Han X. Zhang Z. Joshi J. Borza T. Mohammad Aqa M.. (2020). Exogenous phosphite application alleviates the adverse effects of heat stress and improves thermotolerance of potato (*Solanum tuberosum* L.) seedlings. Ecotoxicol. Environ. Saf. 190, 110048. doi: 10.1016/j.ecoenv.2019.110048 31837570

[B114] Xu D. Xiong T. Lu W. Zhao J. Zhang Z. Xiao G. (2024). The ptxD Gene Confers Rapeseed the Ability to Utilize Phosphite and a Competitive Advantage against Weeds. Agronomy 14 (4), 727. doi: 10.3390/agronomy14040727

[B115] Yang S. Gao X. W. Diao C. L. Song B. A. Jin L. H. Xu G. F.. (2006). Synthesis and antifungal activity of novel chiral α-aminophosphonates containing fluorine moiety. Chin. J. Chem. 24, 1581–1588. doi: 10.1002/cjoc.200690296

[B116] Yang K. Metcalf W. W. (2004). A new activity for an old enzyme Escherichia coli bacterial alkaline phosphatase is a phosphite-dependent hydrogenase. Proc. Natl. Acad. Sci. U.S.A. 101, 919–7924. doi: 10.1073/pnas.0400664101 PMC419532 15148399

[B117] Yuan H. Wang Y. Liu Y. Zhang M. Zou Z. (2021). A novel dominant selection system for plant transgenics based on phosphite metabolism catalyzed by bacterial alkaline phosphatase. PloS One 16 (11), e0259600. doi: 10.1371/journal.pone.0259600 34735551 PMC8568168

[B118] Zambrosi F. C. B. M. J. Syvertsen a.P. (2011). Plant growth, leaf photosynthesis, and nutrient-use efficiency of citrus rootstocks decrease with phosphite supply. J. Plant Nutt. Soil Sci. 174, 487–495. doi: 10.1002/jpln.201000320

[B119] Zheng X. T. Yu Z. C. Tang J. W. Cai M. L. Chen Y. L. Yang C. W.. (2021). The major photoprotective role of anthocyanins in leaves of *Arabidopsis thaliana* under long-term high light treatment: antioxidant or light attenuator? Photosynth. Res. 149, 25–40. doi: 10.1007/s11120-020-00761-8 32462454

